# Cell-Specific Dual Role of Caveolin-1 in Pulmonary Hypertension

**DOI:** 10.1155/2011/573432

**Published:** 2011-05-22

**Authors:** Rajamma Mathew

**Affiliations:** Section of Pediatric Cardiology, Department of Physiology, New York Medical College, Valhalla, NY 10595, USA

## Abstract

A wide variety of cardiopulmonary and systemic diseases are known to lead to pulmonary hypertension (PH). A number of signaling pathways have been implicated in PH; however, the precise mechanism/s leading to PH is not yet clearly understood. Caveolin-1, a membrane scaffolding protein found in a number of cells including endothelial and smooth muscle cells, has been implicated in PH. Loss of endothelial caveolin-1 is reported in clinical and experimental forms of PH. Caveolin-1, also known as a tumor-suppressor factor, interacts with a number of transducing molecules that reside in or are recruited to caveolae, and it inhibits cell proliferative pathways. Not surprisingly, the rescue of endothelial caveolin-1 has been found not only to inhibit the activation of proliferative pathways but also to attenuate PH. Recently, it has emerged that during the progression of PH, enhanced expression of caveolin-1 occurs in smooth muscle cells, where it facilitates cell proliferation, thus contributing to worsening of the disease. This paper summarizes the cell-specific dual role of caveolin-1 in PH.

## 1. Introduction

Pulmonary hypertension (PH) is a rare but a devastating disease with high morbidity and mortality rate. The reported prevalence is 15–52 cases/million and the incidence is thought to be 2.4–7.6 cases/million/year [1, 2]. A wide variety of cardiopulmonary diseases, collagen vascular and autoimmune diseases, chronic thromboembolism, HIV, portal hypertension, drug toxicity, and myeloproliferative diseases are known to lead to PH. In primary pulmonary arterial hypertension (PAH), currently labeled as idiopathic PAH, the underlying etiology is not clear and about 6% of patients in this group have a family history of the disorder [3, 4]. Multiple signaling pathways and inflammation have been implicated in the pathogenesis of PH. Endothelial dysfunction may be an important triggering factor leading to an imbalance between vasorelaxation and vasoconstriction and deregulation of cell proliferation leading to vascular remodeling and PH with subsequent cell migration and neointima formation. Loss of bioavailability of nitric oxide (NO) and prostacyclin (PGI_2_) [5–7], upregulation/activation of proliferative molecules such as endothelin-1 (ET1) [8, 9], platelet-derived growth factor (PDGF) [10], serotonin [11], survivin [12], cyclin D1 [13], tyrosine-phosphorylated signal transducer and activator of transcription 3 (PY-STAT3) [14, 15], RhoA/Rho kinase [16, 17], and anti-apoptotic molecules such as Bcl2 and Bcl-xL [18, 19] have been reported in PH. In addition, increased elastase activity [20] and increased production of matrix metalloproteinase 2 (MMP2) [21] occur in PH. Recent studies have shown a strong link between heterozygous germline mutations in bone morphogenic protein receptor type II (BMPRII), a member of TGF*β* superfamily and pulmonary arterial hypertension (PAH). Mutation of BMPRII has been reported in 70% of heritable PAH, 26% IPAH, and 6% of patients with congenital heart defect and associated PAH. However, only about 20% of people with this mutation develop PAH [22–25], indicating that environmental and/or other genetic factors may be involved in the development of the disease. Furthermore, recent studies have shown reduction in the expression of BMPRII protein in both monocrotaline (MCT) and the hypoxia models of PH [26, 27]. In addition, mutations of activin-like receptor kinase 1 (ALK1) and endoglin, both belonging to TGF*β* superfamily, have been reported in patients with hereditary hemorrhagic telangiectasia, and some of these patients develop PAH [28].

Regardless of the underlying etiology, the main features are endothelial dysfunction, impaired vascular relaxation response, deregulated cell proliferation and impaired apoptosis, vascular remodeling, narrowing of the lumen, elevated PA pressure, and right ventricular hypertrophy with subsequent right heart failure and premature death. Despite major advances in the understanding of the disease process, a cure is not yet in sight. Current therapy has improved the quality of life but has not had a significant effect on the mortality rate [29]. Loss of endothelial caveolin-1, a cell membrane protein is well documented in experimental and clinical forms of PH [13, 14, 30]. Recent studies indicate that in addition to the loss of endothelial caveolin-1, there is enhanced expression of caveolin-1 in smooth muscle cells with proliferative activity and subsequent neointima formation [31, 32]. Thus, caveolin-1 may play a key role in the pathogenesis of PH, and its activity may depend on cell type and the disease stage.

## 2. Caveolin-1 and Caveolae

 Caveolae are 50–100 nm flask-shaped invaginations rich in cholesterol and sphingolipids was described by Palade and Yamada in 1950s [33, 34]. Caveolae are a subset of lipid rafts found on the plasmalemmal membranes of a variety of cells including endothelial, smooth muscle, epithelial cells, and fibroblasts. One of the major functions of caveolae is to serve as a platform and to compartmentalize the signaling molecules that reside in or are recruited to caveolae. Caveolae are also involved in transcytosis, endocytosis, and regulation of cell proliferation, differentiation, and apoptosis via a number of diverse signaling pathways. Three isoforms of caveolin gene family have been identified. Caveolin-3 is a muscle-specific gene found primarily in skeletal and cardiac myocytes. Caveolin-2 not only colocalizes with caveolin-1 but also requires caveolin-1 for membrane localization. Caveolin-1 (22 kD) is the major constitutive protein of caveolae that interacts and regulates several proteins including Src family of kinases, G-proteins (*α* subunits), G protein-coupled receptors, H-Ras, PKC, eNOS, integrins, and growth factor receptors such as VEGF-R and EGF-R. Caveolin-1 stabilizes these signaling proteins, and generally, protein-protein interaction with caveolin-1 exerts negative regulation of the target protein within caveolae; these interactions occur through caveolin-1-scaffolding domain (CSD, residue 82–101 in caveolin-1) [35–41]. Major ion channels such as Ca^2+^-dependent potassium channels and voltage-dependent K^+^ channels (Kv 1.5) and a number of molecules responsible for Ca^2+^  handling such as inositol triphosphate receptor (IP_3_R), heterodimeric GTP-binding protein, Ca^2+^ ATPase, and several transient receptor potential channels localize in caveolae and interact with caveolin-1. In SMC, caveolin-1 regulates Ca^2+^ entry and enables vasoconstriction. The localization of Ca^2+^-regulating proteins in caveolae and the proximity to sarcoplasmic reticulum is indicative of an important role for caveolae/caveolin-1 for Ca^2+^ homeostasis [42–44]. RhoA interacts directly with caveolin-1, and the translocation of RhoA to caveolae is required for myogenic tone. The CSD peptide of caveolin-1 has been shown to inhibit the agonist-induced redistribution of RhoA and PKC-*α* [45–47]. In addition, caveolin-1 ablation attenuates both pressure and agonist-induced vasoconstriction [48]. Thus, caveolae/caveolin-1 plays a complex and important role in regulating Ca^2+^ homeostasis. 

Endothelial cells (EC) are thought to have one of the highest levels of caveolin-1 [36]. One of the major functions of endothelial cells is to maintain vascular tone and structure; in addition, endothelial cell layer forms an important interface between circulating blood and vascular smooth muscle cells. Nitric oxide (NO), endothelium-derived hyperpolarizing factor (EDHF), and PGI_2_ are potent vasodilators and antimitogenic factors produced by endothelial cells to maintain vascular health. Sustained Ca^2+^ entry into EC regulated by caveolin-1 is required for the activation of NO, PGI2, and EDHF. Importantly, caveolin-1 deficiency impairs Ca^2+^ entry, thus caveolin-1 has an impact on the activation of these vasoactive factors [49]. Endothelial NO synthase (eNOS) catalyzes conversion of L-arginine to NO. For optimum activation, eNOS is targeted to caveolae. Although it is negatively regulated by caveolin-1, caveolin-1 is crucial for NO-mediated angiogenesis [50–54]. In addition, the downstream effector of NO, soluble guanylate cyclase has been shown to compartmentalize in caveolae to facilitate its activation [55]. Genetic deletion of caveolin-1 has been shown to abrogate EDHF-induced hyperpolarization by altering Ca^2+^ entry, thus highlighting the role of caveolin-1 in EDHF regulation [56]. PGI_2_, a potent vasodilator produced by EC is formed from arachidonic acid by the enzymatic activity of PGI_2_ synthase, which also colocalizes with caveolin-1. Unlike eNOS, PGI_2 _synthase remains enzymatically active even when bound to caveolin-1. Colocalization of eNOS and PGI_2_ synthase and VEGFR2 with caveolin-1 suggests a role for caveolin-1 in angiogenesis signaling pathways [57]. Furthermore, VEGFR2 activation has been shown to be impaired in endothelial cells from caveolin-1 knockout (KO) mice, further supporting a role for caveolin-1 in VEGF-induced signaling [52, 58]. 

Recent studies have shown that caveolae and caveolin-1 may serve as mechanosensers and/or transducers in arterial responses to alterations in blood flow. Vascular endothelial cells subjected to blood flow-induced shear stress transform mechanical stimuli into biological signaling known as mechanotransduction. Mechanosensing by endothelial cells results in the activation of MAPK/ERK, Akt, and eNOS pathways, thus preventing apoptosis and facilitating vasodilation. Furthermore, impaired flow-mediated vasoldilation in caveolin-1 KO mice is rescued by reconstitution of caveolin-1. Interestingly, poor activation of ERK 1/2 and Akt in EC with low caveolin-1 is thought to be due to impaired VEGFR2 signaling [59, 60]. Thus, caveolin-1 and caveolae are essential for maintaining normal function of blood vessels. 

Caveolin-1, also known as a tumor-suppressor factor, inhibits a number of proliferative pathways. Downregulation of caveolin-1 has been shown to lead to hyperactivation of MAPK/ERK pathway. In caveolin-1 KO mice, observed cell hyperproliferation has been attributed to MAPK/ERK signaling pathway. Interestingly, caveolin-1 is negatively regulated by MAPK/ERK pathway [61–63]. Caveolin-1 regulates apoptosis in several cell types. It promotes cell-cycle arrest via a *p*53/*p*21^waf1/cip1^-dependent mechanism. It has been further shown that caveolin-1 facilitates apoptosis via suppressing survivin [64–66]. Caveolin-1 inhibits PDGF receptor signaling and is capable of transforming PDGF-induced proliferative signals into death signals [67, 68]. Caveolin-1 also functions as an immunomodulator. It modulates inflammatory processes via its regulatory effect on eNOS, and depending on the cell type and possibly the disease, the effect can be positive or negative. In addition, caveolin-1 inhibits and degrades inflammatory and proneoplastic protein COX2 to maintain it at the homeostatic level, and caveolin-1 regulation of TGF-*β* signaling and modulates several cellular processes including differentiation and migration. TGF-*β*1 receptor activin like receptor kinase (ALK) 1 colocalizes with caveolin-1 and both proteins regulate angiogenesis [69–76]. 

 Thus, caveolin-1 regulates and interacts directly or indirectly with a number of signaling molecules implicated in PH. Therefore, there is a reason to believe that the alterations in endothelial cell membrane integrity and caveolin-1 may have a profound effect on the development and the progression of pulmonary vascular disease.

## 3. Endothelial Caveolin-1 in Pulmonary Hypertension

### 3.1. Disruption of Endothelial Cell Membrane and Loss of Caveolin-1

Loss of endothelial caveolin-1 has been reported in clinical and experimental forms such as monocrotaline (MCT) and myocardial infarction models of PH [13, 14, 30]. The MCT model has been extensively studied to understand the pathogenesis of PH. A single subcutaneous injection of MCT in rats injures endothelial cells within 24–48 hrs and PH is observed at 10–14 days after MCT. In this model the disruption of endothelial caveolae associated with progressive loss of caveolin-1 occurring as early as 48 hrs after MCT, is a major feature seen before the onset of PH. In addition to the loss of caveolin-1, there is reduction in the expression of other endothelial cell membrane proteins known to colocalize with caveolin-1 such as Tie2 (endothelium-specific tyrosine kinase receptor of angiopoietin 1), platelet endothelial cell adhesion molecule (PECAM) 1, and both subunits of soluble guanylate cyclase [14, 19]. Importantly, the loss of caveolin-1 is associated with reciprocal activation of signal transducer and activator of transcription (STAT) 3 to PY-STAT3, known to be preferentially activated by downstream effectors of proinflammatory cytokine IL-6/gp130 signaling pathway. In addition, the expression of Bcl-xL is increased simultaneously with the activation of PY-STAT3. PY-STAT3 plays a critical role in cell growth, inhibition of apoptosis, survival, and in immune function and inflammation. Persistent phosphorylation of STAT3 has been reported in a number of primary tumors, and activation of STAT3 signaling confers resistance to apoptosis, [13, 14, 19, 77]. Some of the downstream effectors of PY-STAT3 are survivin and Bcl-xL (antiapoptotic factors), and cyclin D1 (cell-cycle regulator). All these factors have been shown to be upregulated in PH. Importantly, activation of PY-STAT3 has been observed in endothelial cells obtained from patients with idiopathic PAH [15]. RhoA/Rho kinase activation is well established in PH, and interestingly, Rho GTPases is required for STAT3 activation, and Rho GTPases-mediated cell proliferation and migration occur via STAT3 [17, 78]. Caveolin-1 functions as a suppressor of cytokine signaling (SOCS) 3 and inhibits PY-STAT3 activation [79]. Therefore, it is not surprising that the rescue of endothelial caveolin-1 not only inhibits STAT3 activation but also restores the endothelial cell membrane integrity and attenuates MCT-induced PH and vascular remodeling [80–82]. These results underscore the importance of endothelial cell membrane integrity and the expression of endothelial caveolin-1 in maintaining vascular health. 

Studies with caveolin-2 KO mice have shown pulmonary defects such as alveolar wall thickening and increased cell proliferation similar to what has been reported in caveolin-1 KO mice. Unlike caveolin-1 KO, caveolin-2 KO has no effect on vascular reactivity, nor does it participate in the formation of caveolae. Interestingly, in the MCT and myocardial infarction models of PH, in addition to loss of caveolin-1, caveolin-2 loss occurs, and the rescue of caveolin-1 attenuates PH and also restores caveolin-2 expression [13, 81, 83]. Since caveolin-2 requires caveolin-1 for its transport to the membrane surface, caveolin-2 loss may accompany the caveolin-1 loss in these models of PH. It is likely that caveolin-2 participates with caveolin-1 in pulmonary vascular health and disease. It is not clear what independent role caveolin-2 might have in the pathogenesis of PH. Further studies are warranted to examine the specific role of caveolin-2 in PH.

### 3.2. Perturbation of Endothelial Cell Membrane and Dysfunction of Caveolin-1

PH is an important cause of heart failure and increased mortality in patients suffering from chronic lung diseases associated with alveolar hypoxia [84]. Hypoxia induces pulmonary vasoconstriction and vascular remodeling leading to PH. In hypoxia-induced PH, similar to the MCT model, low bioavailability of NO, low basal and agonist-induced cGMP levels, and impaired endothelium-dependent NO-mediated relaxation responses in pulmonary arteries have been reported [85–87]. Interestingly, BH4 or L-arginine administration does not improve eNOS dysfunction [85]. However, unlike the MCT model, in hypoxia-induced PH, there is no reduction in caveolin-1 expression. Murata et al. [85] have further shown that in pulmonary arteries from rats with hypoxia-induced PH, eNOS forms a tight complex with caveolin-1 and becomes dissociated from HSP90 and calmodulin, resulting in eNOS dysfunction. In addition, the long-term effect of prenatal hypoxia results in impaired endothelium-dependent and NO-mediated relaxation responses coupled with increased caveolin-1 and eNOS association [88]. Interestingly, hypoxia-induced PH and pulmonary endothelial cells exposed to hypoxia exhibit hyperactivation of PY-STAT3 [89]. Hypoxia-inducible factor (HIF) 1*α* is thought to play a significant role in hypoxia-induced hyperplasia of SMC [90]. STAT3 plays a significant role in stabilizing HIF1*α*, and its interaction with HIF1*α* mediates transcriptional activation of vascular endothelial growth factor (VEGF) promoter. Targeting STAT3 blocks HIF1*α* and VEGF, thus modulating proliferation and angiogenesis [91, 92]. These results strongly suggest that PY-STAT3 may be an important regulator of VSMC proliferation in PH irrespective of the underlying etiology.

**Figure 1:**
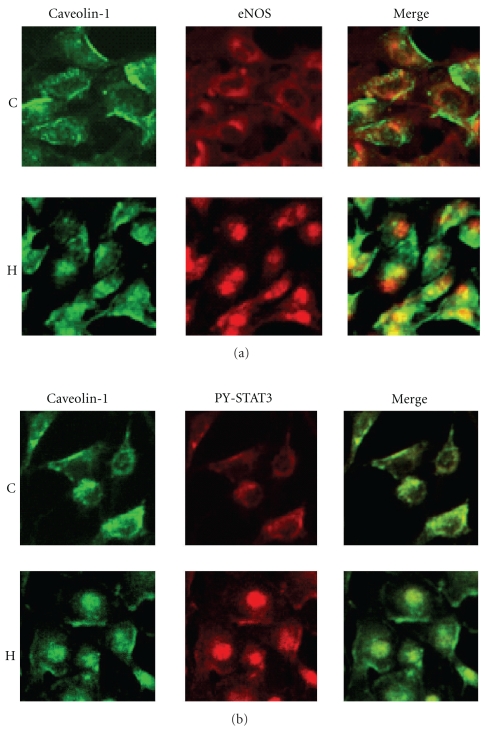
Bovine pulmonary artery endothelial cells (BPAEC) after 24 hr exposure to hypoxia (H, 5% O_2_) and in normoxia (C). (a) During hypoxia the expression of caveolin-1 (green) and eNOS (red) appear increased and they form a complex. (b) PY-STAT3 (red) activation is observed after exposure to hypoxia.

Since caveolin-1 has been shown to inhibit PY-STAT3 activation [79–81], the activation of PY-STAT3 in hypoxia-induced PH despite the unaltered expression of caveolin-1 protein strongly suggests that caveolin-1 is dysfunctional and has lost its inhibitory function. Furthermore, within 24 hr exposure to hypoxia, bovine pulmonary artery endothelial cells reveal caveolin-1 and eNOS complex formation accompanied by PY-STAT3 activation (Figures 1(a) and 1(b)). These results indicate that the tight complex formation of caveolin-1 and eNOS in hypoxia-induced PH renders both eNOS and caveolin-1 dysfunctional. In this context, it is worth noting that statins protect eNOS function in hypoxia-induced PH. The major effect of statins is reported to be the uncoupling of eNOS/caveolin-1 complex, thus freeing eNOS for activation. This effect on eNOS is not accompanied with lowering of cholesterol [93]. It is likely that the statins disrupt the tight cavolin-1/eNOS coupling resulting from hypoxia- induced perturbation of endothelial cell membrane, thus restoring antiproliferative properties of caveolin-1 and NO production by eNOS. Unlike the MCT model, hypoxia does not appear to cause physical disruption of EC membrane but causes perturbation of the endothelial cell membrane and leading to “mislocalization” of caveolin-1 and eNOS.

## 4. Progressive Endothelial Cell Damage and Enhanced Expression of Caveolin-1 in SMC in Pulmonary Hypertension

Juxtaposition of EC and SMC facilitates crosstalk and co-regulation and EC protects SMC from blood elements and direct shear stress. Our studies with experimental and clinical PH show that inflammation and drug-induced injury disrupt endothelial cell membrane integrity leading to the loss of endothelial caveolin-1. The endothelial damage is progressive resulting in the loss of cytosolic proteins, such as HSP90, Akt, I*κ*B*α*, eNOS and subsequently the loss of von Willebrand factor (vWF) [14, 19, 32]. vWF, synthesized by endothelial cells is stored in Weibel Palade bodies within the cell; therefore, the loss of vWF is indicative of an extensive endothelial damage or loss. Importantly, increased plasma levels of vWF and circulating endothelial cells are markers of poor prognosis in PAH [18, 94, 95]. Enhanced expression of caveolin-1 in SMC is seen only in the arteries exhibiting vWF loss [32], and interestingly, a loss/apoptosis of endothelial cells is thought to trigger SMC proliferation [96]. 

The number of caveolae in SMC are said to be less than half that of EC [97]. Recent studies have shown robust expression of caveolin-1 in SMC in PAH, in addition to the loss of endothelial caveolin-1 [31, 32]. Following immunosuppressant therapy-induced endothelial damage, a significant loss of endothelial caveolin-1 and vWF was shown to be associated with robust expression of caveolin-1 in SMC with subsequent neointima formation [32]. Interestingly, in patients with chronic obstructive pulmonary disease, expression of caveolin-1 in SMC was seen only in the presence of PH [98]. Smooth muscle cells isolated from patients with idiopathic PAH exhibit not only enhanced caveolin-1 expression but also altered Ca^2+^ handling, increased cytosolic [Ca^2+^]_i_ and increased DNA synthesis. Increased [Ca^2+^]_i_ is a trigger for DNA synthesis and cell proliferation [31]. Caveolin-1 is considered to play an important role in the regulation of vascular SMC in receptor-mediated signaling [99]. Caveolin-1 regulates mitogenic signaling and Ca^2+^ entry in SMC. Normally, caveolin-1 keeps mitogens inactive in caveolae, but under increased mechanical stress/strain, caveolin-1 translocates from caveolae to noncaveolar sites within the plasma membrane of cultured SMC and translocated caveolin-1 triggers cell-cycle progression and cell proliferation [100, 101]. The extensive endothelial damage, as described in the preceding section, may result in a breach in the endothelial layer, thus exposing SMC to high cyclic pressure with consequent enhanced expression of caveolin-1 in SMC and possible translocation from caveolae. Interestingly, caveolin-1 requires cavin-1 (one of the cavin protein family also known as polymerase 1 and transcript release factor) for caveolae formation. Recent *in vitro* studies show that cells (murine lung endothelial and HeLa cells) exposed to mechanical stress reveal reduced caveolin-1/cavin-1 interaction, disappearance of caveolae, and increased expression of caveolin-1 at the plasma membrane [102, 103]. It is tempting to postulate that in PH, the observed enhanced expression of caveolin-1 in SMC may in part be related to the reduction in caveolin-1/cavin-1 interaction and loss of caveolae during exposure to pulsatile shear stress. Further studies are required to address caveolin-1/cavin-1 interaction in SMC in PH. 

 SMC migration, matrix degradation, and remodeling leading to neointima formation are essential aspects of PH. Matrix metalloproteinases (MMP); especially, the activation of MMP2 is considered a critical step in the migration of SMC through the basement barrier. MMP2 belongs to a family of zinc-dependent endopeptidases called MMPs, involved in matrix degrading processes. MMP2 has been implicated in vascular remodeling and plays an important role in cell migration, thus contributing to neointima formation. Not surprisingly, increased expression and activity of smooth muscle cell MMP2 has been reported in idiopathic PAH. Importantly, MMP2 and its physiologic activator MT1-MMP colocalize in caveolae and are negatively regulated by caveolin-1 [21, 104–106]. This suggests that the exposure of SMC to increased shear stress may translocate caveolin-1 from caveolae to other plasma membrane sites, thus losing its inhibitory activity on MT1-MMP and MMP2 and facilitating cell migration via MMP2. In support of this view, it has been shown that the caveolin-1 re-expression in tumor cells inhibited MMP activation and function thereby preventing cell migration [107]. In vascular SMC, the localization of BMPRII to caveolae and binding with caveolin-1, required for efficient signal transduction and disruption of caveolin-1 and BMPRII interaction negatively affects BMP2-dependent SMAD phosphorylation [108]. Thus, the localization of caveolin-1 in caveolae may be of paramount importance for its activity.

Acute respiratory syndrome (ARDS) is comprised of increased pulmonary vascular permeability and pulmonary edema associated with hypoxemia and carbon dioxide retention, caused by a variety of pulmonary and systemic insults and drug toxicity. Endothelial injury is a critical underlying feature resulting in the disruption of alveolar-capillary membrane and vascular fluid leak. The reported incidence is about 2.2–16/100,000 with a mortality rate of about 40%. Incidence of PH is as high as 92% in patients with ARDS, severe and moderated being 7% and 76% respectively [109–111]. A recent case report [32] has shown that within 2 months of developing ARDS, lung biopsy revealed loss of endothelial caveolin-1 in several arteries without any evidence of PH. This is similar to the observation in the MCT model, where the progressive loss of endothelial caveolin-1 occurs before the onset of PH [19, 80]. The second patient developed severe PH despite having clinically recovered from ARDS two years earlier. Importantly, the loss of vWF was associated with robust expression of caveolin-1 in SMC with subsequent neointima formation [32]. Interestingly, increased plasma vWF antigen is an independent marker of poor prognosis in acute lung injury and is associated with high mortality, similar to what has been reported in PAH [95, 112]. Thus, the loss of endothelial cell membrane integrity, and resulting caveolin-1 loss, directly or indirectly facilitates vascular leak and has the potential to initiate pulmonary vascular disease. Progressive endothelial damage as indicated by loss of vWF and enhanced expression of caveolin-1 in SMC may portend worsening PH. 

It is worth noting that caveolin-1, a marker of mature and contractile SMC, keeps SMC in quiescence. It modulates Ca^2+^-regulatory molecules, increases Ca^2+^ mobilization and facilitates contractile responses to agonists. In addition, interaction of caveolin-1 and RhoA is critical for pressure-induced myogenic tone in resistance arteries. Therefore, it is not surprising that the arteries from caveolin-1 KO mice develop poor myogenic tone in response to pressure. Caveolin-1 also prevents proliferative activity. It inhibits receptor and nonreceptor tyrosine kinases by sequestering them to caveolae and prevents cell proliferation. Furthermore, disruption or ablation of caveolin-1 in airway and vascular SMC show increased cell proliferation [113–115]. Thus, caveolin-1 is essential for normal functioning of SMC. 

**Figure 2:**
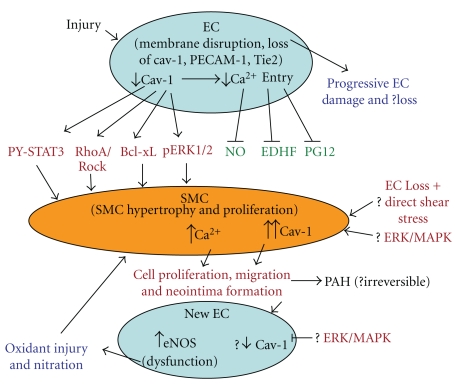
This figure depicts the proposed model of PH (#1). Injury to EC results in progressive loss of endothelial caveolin-1, loss of vasodilators, and the activation of proliferative and anti-apoptotic pathways leading to PH. EC disruption is progressive resulting in further loss of multiple proteins and possibly the loss of EC. Extensive damage/loss of EC exposes SMC to direct shear stress leading to enhanced expression of caveolin-1 which participates in further cell proliferation and cell migration resulting in neointima formation. Newly formed EC in neointima express increased eNOS; possibly low caveolin-1 expression in these cells may in part be responsible for the observed dysfunctional eNOS. The resulting oxidant/nitration injury further influences PH adversely.

**Figure 3:**
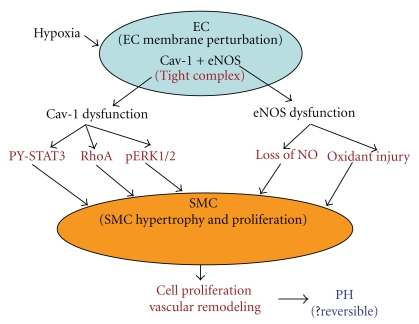
This figure depicts the proposed model of PH (#2). Hypoxia causes perturbation of endothelial cell membrane, and caveolin-1 and eNOS form a tight complex rendering both molecules dysfunctional. eNOS dysfunction function leads to impaired NO availability and superoxide generation, and dysfunctional caveolin-1 loses its ability to inhibit proliferative pathways resulting in the activation of mitogenic pathways. The end results are pulmonary vascular remodeling and PH.

 Taken together, our studies and published reports, it appears that injury to EC (shear stress, drug toxicity, inflammation) results in (a) a progressive loss of endothelial caveolin-1, (b) impaired Ca^2+^entry into EC resulting in reduced production of vasodilators, and (c) reciprocal activation of mitogenic signaling pathways leading to vascular cell proliferation, medial wall thickening, and PH. It is possible that PH at this stage may be reversible. Progressive EC damage results in further loss of proteins including eNOS. As the disease progresses, extensive EC damage/loss occurs followed by enhanced expression of cav-1 in SMC, where cav-1 facilitates cell proliferation and migration leading to neointima formation. It is likely that translocated caveolin-1 in SMC not only loses its ability to inhibit proliferative pathways but also switches from being antiproliferative to proproliferative that may eventually lead to SMC phenotype change from contractile to synthetic type. Recent studies indicate that there is increased expression of eNOS in neointimal EC, but eNOS is dysfunctional, resulting in oxidant/nitration injury, thus further aggravating PH as outlined in Figure 2. In contrast, perturbation of EC in hypoxia model of PH results in a tight complex formation of caveolin-1 and eNOS leading to dysfunction of both caveolin-1 and eNOS. The end results are impaired availability of NO, the activation of proliferative pathways, vascular remodeling, and PH. It is worth noting that there is no loss of eNOS or caveolin-1 protein in this model (Figure 3).

## 5. Caveolin-1 Knockout (KO) Mice and Pulmonary Hypertension

Studies conducted with caveolin-1 KO mice have provided valuable information regarding the function of caveolin-1 in pulmonary vascular health and disease. Caveolin-1 KO mice are viable and fertile, but with significantly shortened life span. Embryonic fibroblasts taken from these animals show increased capacity for cell proliferation, and arterial SMC lacking caveolin-1 display abnormalities in proliferation both in vivo and in vitro. Furthermore, these mice manifest hypercellular lung phenotypes, cardiomyopathy, systemic vasculopathy, and a propensity to develop PH [115–120]. In caveolin-1 KO mice there is a global loss of caveolin-1 associated with hyperactivation of eNOS, increased cGMP production, and re-expression of endothelial caveolin-1 restores vascular and cardiac pathology and dysfunction [121]. However, there is a distinct difference between caveolin-1KO mice model of PH and the MCT model vis-à-vis eNOS expression and function. In the latter model, MCT injures pulmonary endothelial cells within the first pass through the lungs resulting in progressive disruption of endothelial cell membrane and a loss of endothelial caveolin-1 with subsequent endothelial dysfunction, vascular remodeling, and PH. At 2 weeks after MCT with the onset of PH, there is impaired endothelium-dependent, NO-mediated pulmonary vascular relaxation response. At this stage, there is a loss of cytosolic proteins such as HSP90 and Akt, both required for eNOS activation. However, eNOS protein expression is relatively well preserved, but there is transient uncoupling of eNOS as indicated by increased superoxide generation, reduction in sulfhydril levels, cGMP levels, and impaired endothelium-dependent NO-mediated vascular relaxation responses [9, 79, 85]. By 3 and 4 wks after MCT, the level of eNOS protein is significantly reduced and the superoxide generation returns to normal levels [9, 19, 122]. 

 It has recently been suggested that the hyperactivation of eNOS subsequently leading to PKG nitration may be responsible for PH in the caveolin-1 KO mice. Interestingly, treatment with superoxide scavenger or the inhibition of eNOS reverses PH, supporting the view [123]. These authors have further shown increased eNOS activation, PKG nitration and reduced caveolin-1 in the lungs from patients with idiopathic PAH [123]. Furthermore, in an earlier report, increased expression of eNOS in plexiform lesions in both primary and secondary PH was described [124]. In contrast, several other studies show loss of eNOS in the lungs from patients with idiopathic PAH, and PH/PAH associate with a variety of diseases such as congenital heart defect, rheumatic heart disease, cirrhosis, and systemic lupus erythematosis [125, 126]. These observed differences in eNOS expression may depend on the stage of disease in a given lung section, because the disease does not progress uniformly. Importantly, it has recently been shown that severe angioproliferative PAH is associated with initial endothelial cell apoptosis followed by the appearance of apoptosis-resistant proliferating endothelial cells [96], and these proliferative EC in neointima have reduced expression of caveolin-1 [30]. Based on these clinical and experimental studies, one could postulate that the initial endothelial injury which is progressive results in extensive endothelial cell damage and/or loss leading to a loss of eNOS protein expression. As the apoptosis-resistant endothelial cells proliferate, increased eNOS expression coupled with low caveolin-1 expression in these cells may lead to eNOS dysfunction. Further studies are required to elucidate these observed differences in the expression of eNOS in PH.

## 6. Nitric Oxide and Pulmonary Hypertension

Impaired NO bioavailability is the hallmark of PH. NO is produced from L-arginine via catalytic activity of eNOS. NO released from EC activates soluble guanylate cyclase to produce cGMP, which facilitates Ca^2+^ uptake into intracellular Ca^2+^ stores, thereby lowering [Ca^2+^]_i_ and inducing vascular relaxation. In addition to the vascular relaxation function, NO/cGMP pathway regulates SMC proliferation and migration, cellular responses to inflammation, controls the concentrations of antioxidants, and modulates apoptosis. Inhibition of NO synthesis results in increased pulmonary vascular thickening and administration of NO attenuates PH. Interestingly, treatment with NO donor started early in the course of MCT-induced PH prevents caveolin-1 disruption and attenuates PH, likely through its anti-inflammatory function [82, 127, 128]. Thus, there is a dynamic interrelationship between eNOS and caveolin-1, and impaired caveolin-1/eNOS interaction and function may be an important factor in the pathogenesis of PH.

## 7. Dual Role of Caveolin-1

Loss of caveolin-1 has been shown to induce oncogenic transformation, and the cells become resistant to apoptosis. Furthermore, the introduction of caveolin-1 scaffolding domain inhibits cancer progression. Many oncogenes transcriptionally downregulate caveolin-1 expression. However, caveolin-1 regulation impacts both negatively and positively on several aspects of tumor progression. Caveolin-1 acts as a tumor suppressor in the early stages of cancer, but in late stages it promotes metastasis, multidrug resistance, and portends poor prognosis. Caveolin-1 function is thought to be interdependent on tumor stage and the expression of molecular effectors that may have an impact on its role during tumor progression [63, 129–131]. Similarly in PH, the switch from an antiproliferative to proproliferative function may depend on alteration in caveolin-1 conformation, localization, cell context, and the stage of the disease.

## 8. Concluding Remarks

Caveolae and caveolin-1 play an important role in pulmonary vascular system. Depending on the type of endothelial injury, the end result is either the loss of caveolin-1 secondary to endothelial cell membrane disruption or in endothelial caveolin-1 dysfunction. A classic example of the latter case is hypoxia-induced PH in which a tight complex formation of caveolin-1/eNOS resulting in dysfunction of both molecules is an important feature. Both these alterations, however, do lead to pulmonary vascular remodeling and PH. Disruption of endothelial cell membrane integrity as in the former case is often progressive leading to extensive EC damage and/or loss with subsequent enhanced expression of caveolin-1 in SMC, which participates in further proliferation, cell migration, and neointima formation. These alterations in caveolin-1 may determine reversibility versus irreversibility of the disease process. Thus, depending on the underlying pathology, cellular involvement, and the stage of the disease, modulation of caveolin-1 function may be considered a therapeutic target in PH.
